# Corneal densitometry after allogeneic small-incision intrastromal lenticule implantation for hyperopia correction

**DOI:** 10.1186/s12886-022-02454-3

**Published:** 2022-06-29

**Authors:** Jie Hou, Yan Wang, Jing Zhang, Yulin Lei, Zhixing Ma, Ying Zhang, Xiuyun Zheng

**Affiliations:** 1 Jinan Mingshui Eye Hospital, Number 5601, Longquan Road, Zhangqiu District, 250200 Jinan, China; 2 Tianjin Key Laboratory of Ophthalmology and Visual Science, Tianjin Eye Hospital, Clinical College of Ophthalmology, Tianjin Medical University, Tianjin, China

**Keywords:** Cornea, Densitometry, Hyperopia, Lenticule implantation, Scheimpflug

## Abstract

**Purpose:**

To evaluate corneal densitometry after allogeneic corneal small-incision intrastromal lenticule implantation (SILI) for hyperopia.

**Methods:**

A retrospective study. Thirty-one hyperopic eyes of 24 patients who underwent SILI were enrolled in this study. Examinations took place preoperatively and 1 week, 1 month, 3 months, and 6 months postoperatively. Corneal densitometry (CD) from different concentric radial zones (0–2, 2–6, and 6–10 mm annulus) and layers (anterior, central, and posterior) were obtained using Scheimpflug imaging. The association between CD changes and the uncorrected distance visual acuity (UDVA), spherical equivalent (SE), central corneal thickness (CCT) and K value were examined.

**Results:**

No serious intraoperative complications occurred during SILI. The mean total CD increased postoperatively compared to preoperatively (*P* < 0.01). However, no significant differences were found among the four subsequent follow-up time points (*P* > 0.05). At 6 months postoperatively, the CD values showed an increase of 2.71 ± 2.52, 2.23 ± 2.25, and 1.87 ± 2.46 at the 0–2, 2–6, and 6–10 mm annuli, respectively (all at *P* < 0.01). The anterior 120 μm displayed the highest densitometry before and after surgery (all at *P* < 0.01). No significant increase was found within the posterior 60 μm of the cornea (*P* > 0.05). No correlation was found between the CD and relevant parameters(all at *P* > 0.05).

**Conclusions:**

SILI resulted in an increase in CD within the surgically altered area, however such change has no significant correlation with visual outcomes.

## Introduction

Small-incision lenticule extraction (SMILE) has been widely used in correction of myopia over the past decades. The stromal lenticule is the immediate by-product of SMILE and can be preserved and reimplanted to correct refractive errors in patients with hyperopia, presbyopia, or to treat keratoconus [1–3]. There are two possible methods to complete the lenticule implantation procedure with femtosecond laser. One is to create a flap and position the lenticule centrally on the optical axis. The second is to make a small incision using the methods of a SMILE procedure followed by insertion of the lenticule into a stromal pocket without the creation of a flap [4]. Previous studies have reported the safety and feasibility of implanting lenticules for the treatment of hyperopia in both human and primates [1, 5–7]. However, the effect intrastromal lenticule implantation may have on corneal transparency has not yet been reported and is important to investigate as it may influence visual outcomes.

Corneal densitometry (CD), which is measured using a Scheimpflug device, is a noninvasive method of quantifying transparency of the cornea by measuring light backscattering in grayscale units. Studies have shown that CD can be used as an objective measure of corneal response after refractive surgery, crosslinking for keratoconus or corneal transplant [8–11]. In the current study, we evaluated the changes in corneal densitometry and it’s effect on visual outcomes after small-incision intrastromal lenticule implantation (SILI) in eyes with hyperopia.

## Methods

### Patients

In this retrospective study, twenty-four patients (31 eyes) with hyperopia who received SILI between October 2018 and May 2020 were enrolled in our study at the corneal refractive surgery center of Jinan Mingshui Eye Hospital. All patients had routine preoperative examinations to exclude surgical contraindications. Inclusion criteria comprised age of 18 to 40 years, spherical refraction from + 2.00 to + 10.00 diopters (D), and astigmatism less than -3.00D. For the myopic patients (donors), astigmatism less than -0.25 D was required. Serology testing was done for hepatitis C virus, hepatitis B virus surface antigen, human immunodeficiency virus and treponema pallidum particle agglutination assay in all donors and recipients before surgery. The study was approved by the Ethical Committee of Jinan Mingshui Eye Hospital, and informed consent was obtained from all patients in accordance with the tenets of the Declaration of Helsinki.

### Surgical techniques

**Fig. 1:**
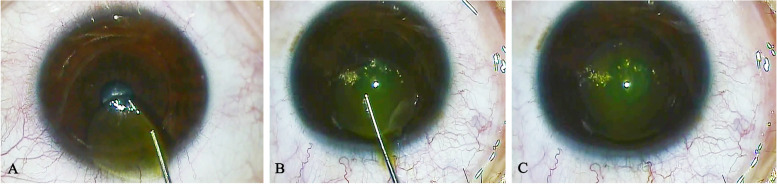
The lenticule implantation procedures. **A** The stained donor lenticule by riboflavin was prepared for the re-implantation. **B** The lenticule was inserted into the pocket with forceps. **C** Lenticular center was aligned with the visual axis

The same surgeon (YL) performed all surgeries. A myopic donor eye was matched with a recipient hyperopic eye that was to be treated with SMILE using VisuMax femtosecond laser system (Carl Zeiss Meditec) on the same day. The thickness of the corneal cap was 120 μm and the diameter of the lenticule was 6.5 mm. The lenticule was extracted and prepared for re-implantation (lenticule refraction = hyperopic eye refraction + 0.50D). The recipient’s eye with hyperopia was firstly treated with SMILE to remove -0.50DS and any corresponding astigmatism. The cap thickness was 120 μm with an intended diameter of 7.3 mm, whereas the lenticule diameter was 6.5 mm. The incision was located superiorly with a length of 4.0 mm. The donor lenticule was soaked in 0.22% riboflavin with saline (VibeX Xtra) for 90 s, then irrigated with saline to rid the excess riboflavin. The stained lenticule was then inserted into the pocket with forceps immediately. After insertion, the lenticule was spread out inside the stromal pocket, and the center was aligned with the visual axis carefully (Fig. 1). A bandage soft contact lens was applied for one day. All patients were instructed to use antibiotics drops for one week and corticosteroid drops for 8 weeks.

### Corneal densitometry

Corneal densitometry was obtained using a device with Scheimpflug imaging (Pentacam, Oculus). The CD data are recorded at 4 annular concentric zones (0–2 mm, 2–6 mm, 6–10 mm and 10–12 mm annulus) centered over the apex of the cornea. The data are also divided into 3 different layers of the cornea: the anterior 120 μm as first layer and the posterior 60 μm as third layer, and the central layer represents the volume between these boundaries. The total measurement represents the average density over the complete corneal thickness. Corneal densitometry is expressed in gray scale units and ranges from 0 (maximum transparency) to 100 (minimum transparency). The 10 to 12 mm annulus data were excluded from the analysis because they were unaffected by SMILE treatment and unreliable in the periphery [12].

### Follow-up

Patients were observed pre-operatively and 1 week, 1 month, 3 months and 6 months postoperatively. UDVA and SE were recorded. Slit-lamp microscopy, Pentacam corneal topography, and optical coherence tomography (OCT) were performed to check for corneal transparency, curvature and thickness.

### Statistical analyses

All statistical analyses were performed using SPSS software (version 20, IBM Corp.). The Kolmogorov–Smirnov test was used to assess the normality of the distribution of all variables. The CD values at different measurement annulus or depths and different time points were compared by repeated-measures analysis of variance. The correlations between the corneal densitometry and relevant parameters were evaluated by using Pearson correlation test. Data were expressed as mean ± standard deviation. A *P* value less than 0.05 was considered statistically significant.

## Results

The median age of all patients at the time of surgery was 20 years old (interquartile ranged from 18 to 21). The mean SE refraction was + 5.95 ± 2.1D (range: + 2.12 to 10.5D) and the mean preoperative CCT measured by OCT was 538.52 ± 30.84 μm (range: 483 to 609 μm). All surgeries were successfully completed. None of the eyes showed evidence of rejection or other serious complications at the last follow-up period.

### Visual outcomes and refraction

**Fig. 2:**
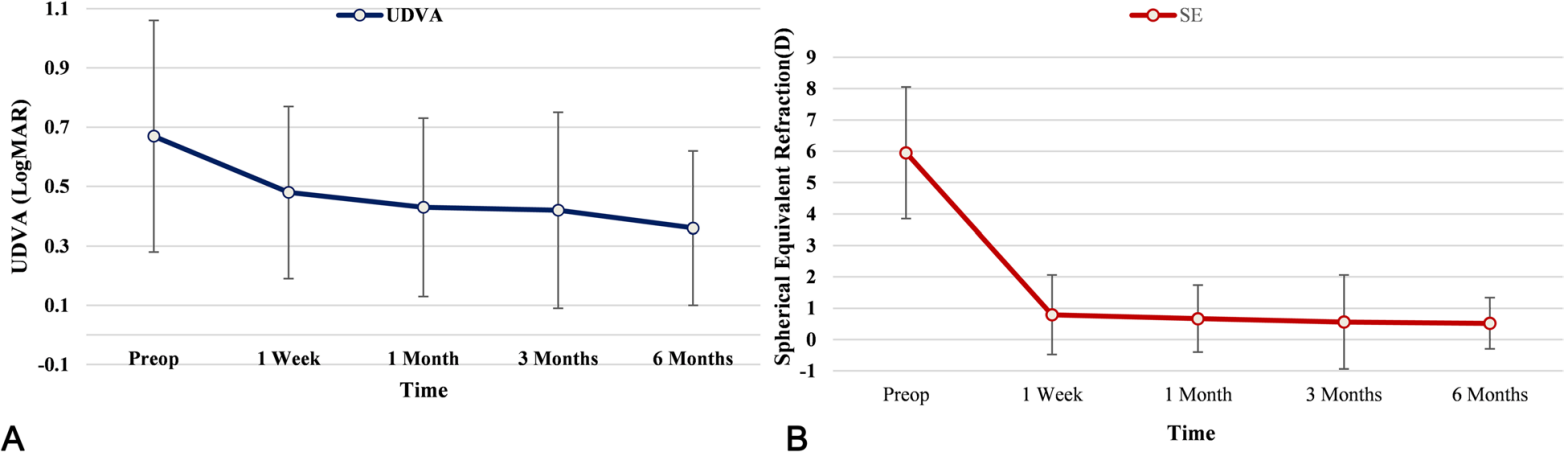
The change of UDVA (LogMAR) and SE before and at 1 week, 1, 3 and 6 months after SILI

The preoperative UDVA (LogMAR) was 0.67 ± 0.39. It was significantly improved and the SE was significantly decreased at each time point after surgery (all at *P* < 0.05). Twenty-eight eyes (90.3%) had an UDVA equal to or better than the preoperative corrected visual acuity at the last follow-up visit. Twelve eyes (38.7%) gained one or two lines. Three eyes (9.7%) lost lines due to the residual refractive error. Twenty-four eyes (77.4%) were within ± 1.00 D of the intended refractive target. No significant difference was found (*P* > 0.05) in SE at 1 week postoperatively compared to the last follow up visit (Table 1, Fig. 2).

**Table 1:** Demographic data pre- and post-surgery

	Pre-	1 week (*n* = 31)	1 month (*n* = 31)	3 month (*n* = 28)	6 month (*n* = 21)	*F* value	*P* value
UDVA	0.67 ± 0.39	0.48 ± 0.29	0.43 ± 0.30	0.42 ± 0.33	0.36 ± 0.26	22.346	< .001^*^
SE	5.95 ± 2.1	0.79 ± 1.27	0.67 ± 1.07	0.56 ± 1.50	0.52 ± 0.82	96.583	< .001^*^
CCT	538.52 ± 30.84	603.35 ± 35.70	606.65 ± 36.0	607.11 ± 35.31	607.14 ± 32.28	81.340	< .001^*^
CET	54.42 ± 2.51	50.23 ± 4.31	49.32 ± 3.22	49.07 ± 3.50	48.76 ± 3.16	23.740	< .001^*^
Km	42.94 ± 1.44	45.86 ± 3.15	46.96 ± 2.61	47.18 ± 2.60	46.75 ± 2.61	39.968	< .001^*^

*UDVA* uncorrected distance visual acuity, *SE* spherical equivalent, *CCT* central corneal thickness, *CET* corneal epithelial thickness, *Km* K value

^*^Significantly different at *P* < .01

### Slit-lamp examination and OCT assessment

A slit-lamp examination was performed to check for the lenticule clarity. The edge of the lenticule was clear and the density of the anterior stroma under the cap was increased at one week and one month after surgery. Alternatively, the outline was almost visible at the next follow-up point. Two eyes had mild opacification one month after surgery, however, both recovered transparency by six months postoperatively.

**Fig. 3:**
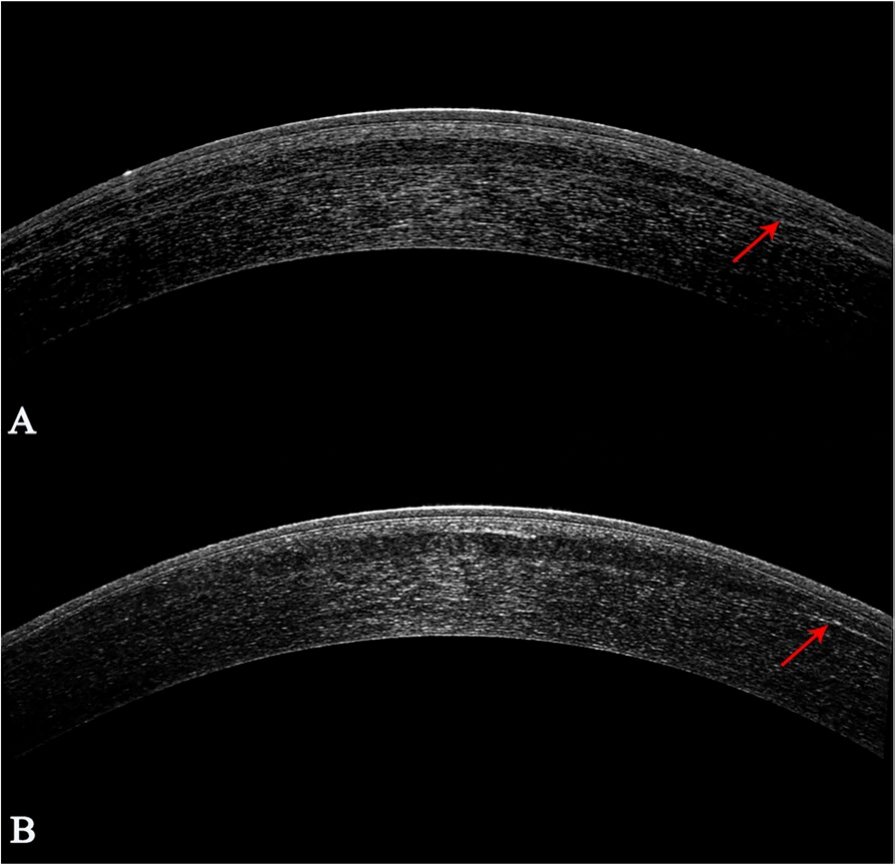
OCT images following SILI. The lenticule contour profile was clearly detected following lenticule implantation. **A** One week postoperatively the lenticule was transparent and the edge was clear. **B** Six months postoperatively the central lenticule was transparent and the demarcation line was blurred, however the density of peripheral tissue increased

At each follow-up visit, the anterior segment OCT images showed that the lenticule implanted was transparent in the central area and adhered tightly to the adjacent tissue. The anterior and posterior surfaces of the lenticule can be easily detected after surgery, while the demarcation line blurred over time, except for in the periphery tissue (Fig. 3).

### Changes in corneal densitometry

Table 2 shows the CD results as measured by Pentacam before and after surgery. The mean CD values of the total cornea increased remarkably compared to the preoperative status (all at *P* < 0.01). Overall CD was 16.60 ± 1.89 prior to the procedure which increased to 18.19 ± 2.68 six months after SLI (*P* < 0.001). However, no significant differences were found among the four follow-up points (*P* > 0.05).

**Table 2:** Changes in corneal densitometry after SILI with different corneal annular concentric zones and depths

Time	Corneal annulus			Corneal layers			Total
	0-2 mm	2-6 mm	6-10 mm	Anterior	Mid-Stromal	Posterior	
Pre-	16.28 ± 1.91	14.88 ± 1.76	16.57 ± 2.76	22.66 ± 2.84	14.28 ± 1.67	12.85 ± 1.59	16.60 ± 1.89
Post-							
1 week	19.32 ± 2.78	17.14 ± 2.13	17.27 ± 2.73	25.92 ± 3.58	15.66 ± 2.11	13.01 ± 2.05	18.19 ± 2.25
1 month	19.09 ± 3.10	16.77 ± 2.09	17.33 ± 2.49	25.42 ± 3.10	15.67 ± 1.72	13.15 ± 1.71	18.08 ± 1.81
3 months	19.00 ± 2.64	16.93 ± 1.66	17.82 ± 2.56	25.57 ± 3.00	15.82 ± 1.81	13.52 ± 1.55	18.30 ± 1.98
6 months	18.81 ± 3.01	16.90 ± 2.46	17.74 ± 2.95	25.12 ± 4.49	16.03 ± 2.30	13.72 ± 2.55	18.19 ± 2.68
* F* value	10.489	10.158	4.143	7.960	8.735	1.579	7.399
* P* value	< .001^*^	< .001^*^	0.009^*^	< .001^*^	< .001^*^	0.188	< .001^*^

^*^Significantly different at *P* < .01

**Fig. 4:**
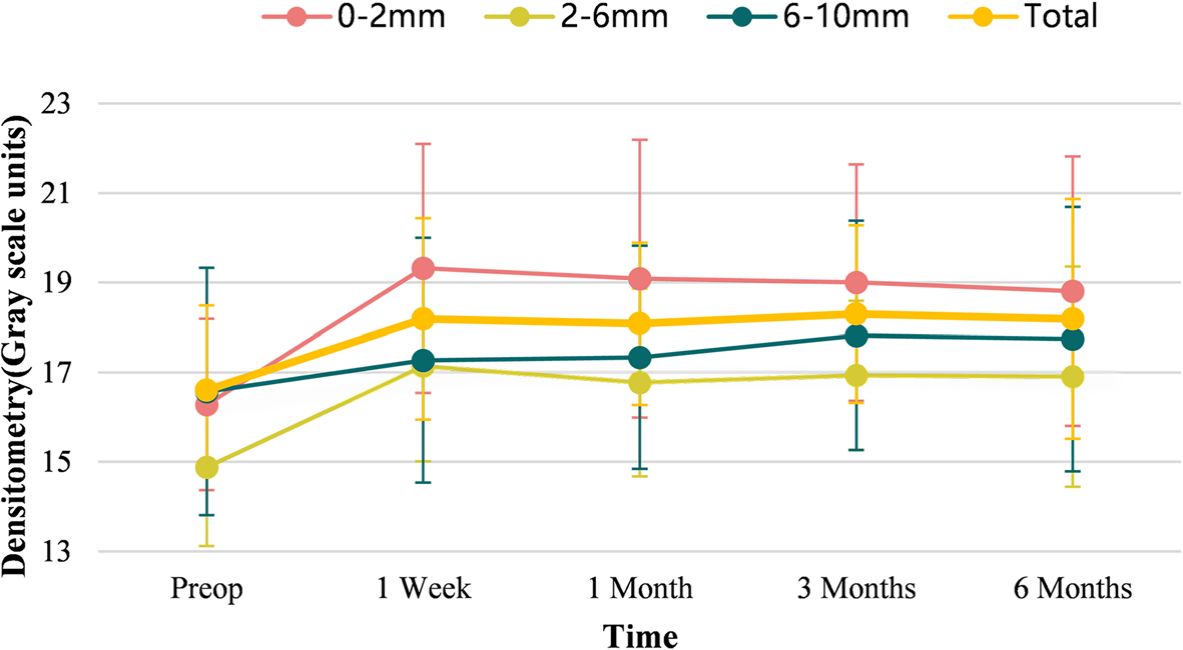
The CD values with different corneal annular concentric zones and the total cornea measured before and at 1 week, 1, 3 and 6 months after SILI

After annular averaging, this trend was also observed at the 0–2 mm, 2–6 mm and 6–10 mm annulus. A significant change in CD was detected among the three annular, and the most pronounced change was observed at the 0–2 mm annulus from one week to three months postoperatively (all *P* values < 0.05). At six months postoperatively, the CD values showed an increase of 2.71 ± 2.52, 2.23 ± 2.25, and 1.87 ± 2.46 at the 0–2 mm, 2–6 mm, and 6–10 mm annulus, respectively (*P* < 0.01). No significant change in CD was detected among the three annulus (*F* = 0.637, *P* = 0.532) (Table 2, Fig. 4).

When divided by depth, Table 2 shows the anterior 120 μm displayed the highest densitometry before and after surgery than that of the other two (*P* < 0.01). The CD of the anterior and central layer of the cornea showed a statistically significant increase postoperatively. At post-operative month six, CD values of the anterior layer and central layer increased by 3.28 ± 3.92, and 2.11 ± 1.91, respectively (*P* < 0.01). No significant increase was found at the posterior layer of the cornea (*P* = 0.188).

No correlations were observed between the CD values and UDVA, SE, CCT, corneal epithelial thickness(CET) and K value at the postoperative examination visit (all at *P* > 0.05).

**Fig. 5:**
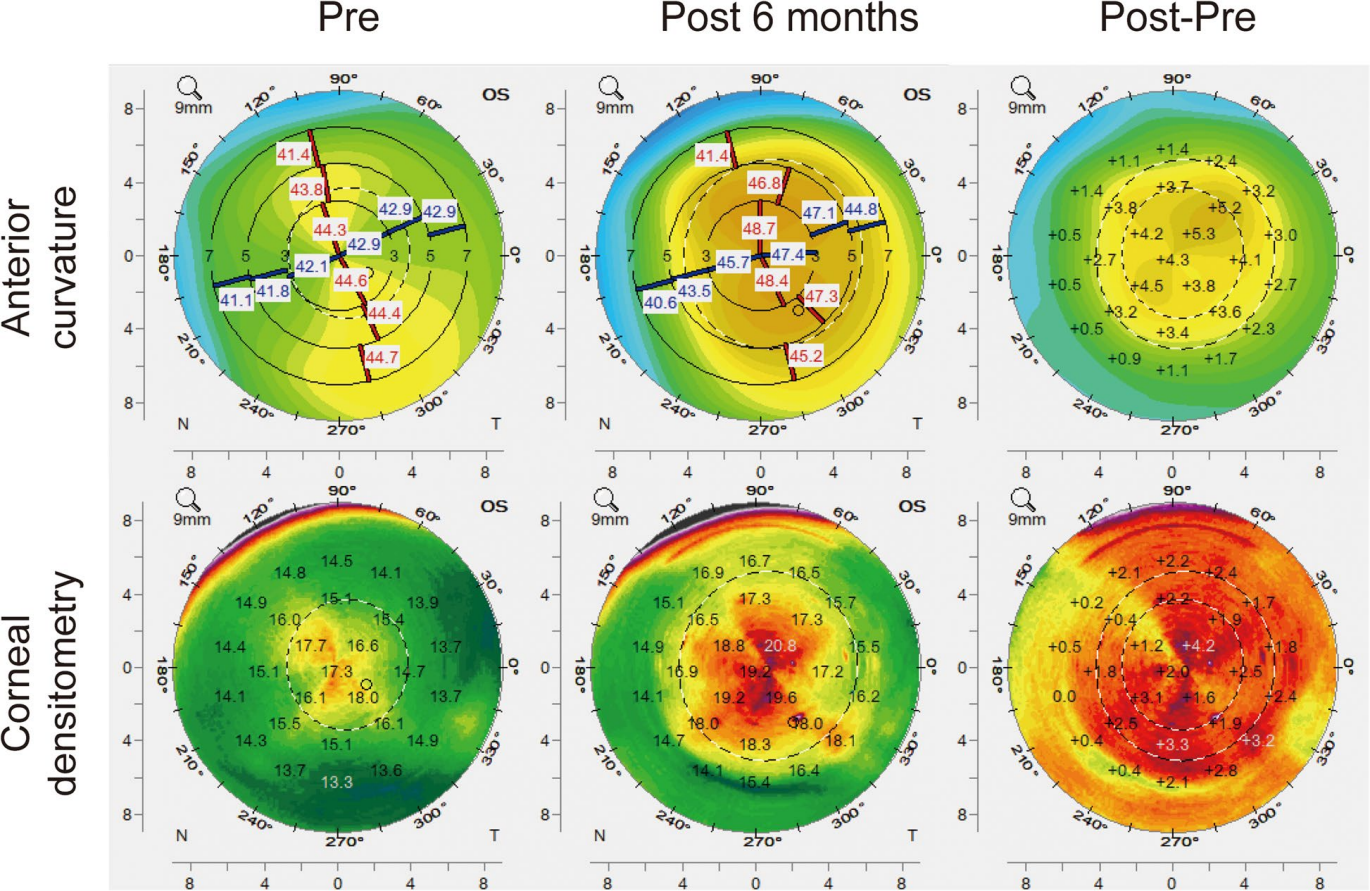
Pentacam topography showing the anterior corneal sagittal curvature and corneal densitometry over the 6-month postoperative course

Figure 5 shows a case of 18-year-old man operated for +6D hyperopia in the left eye before and 6 months after FILI. Corrected distance visual acuity (CDVA) (LogMAR) was 0.5 preoperatively. The UDVA was 0.3 and the SE refraction was + 0.50D 6 month postoperatively. Difference maps displayed a uniform curvature change with good centration(top right) and a significantly corneal densitometry change (bottom right) before and 6 months after surgery.

## Discussion

In small-incision intrastromal lenticule implantation, the lenticule is inserted into a stromal pocket via small-incision without flap creation. It offers certain advantages, including less weakening of corneal biomechanics, less dry eye symptoms, less risk for epithelial ingrowth, less injury to the subbasal nerve plexus and less flap-related complications [4, 5, 13, 14]. In the present study, fresh intrastromal lenticules extracted from SMILE performed in myopic eyes were immediately transplanted into hyperopic eyes using a 4 mm corneal incision. Before allogeneic lenticule implantation, both the donor and recipient received related blood tests to ensure immunologic compatibility for the safety of the surgery. None of the eyes showed evidence of rejection at the last follow-up period of six months.

Pradhan et al. first reported a case of allogeneic lenticule implantation for the correction of high hyperopia and showed that no adverse side effects were observed one year after surgery, but patients only attained 50% of the intended correction [5]. Our results on visual outcomes showed that most eyes gained lines of visual acuity at the last follow-up visit when compared to preoperative values. Seventy-seven percent of patients were within the intended refractive target of ± 1.00 D. Similarly, good refractive predictability after autologous lenticule implantation was observed previously by Li et al. and Ganesh et al. [15, 16]. There were no significant differences in UDVA, SE, K value and CCT when comparing values at 1 week versus 6 months postoperatively. This indicates that SILI provided corrective stability after hyperopic treatment.

The methods of evaluating corneal transparency include slit-lamp microscopy, anterior segment OCT, Scheimpflug CD and in vivo confocal microscopy (IVCM) [17, 18]. In the current study, two eyes had mild haze within the early post-operative period. However, the majority of the lenticule observed via slit-lamp examination remained transparent at each follow-up visits and the lenticule demarcation line blurred over time when viewed on anterior segment OCT.

Monitoring of CD changes can help to evaluate the corneal transparency quantitatively over time after corneal refractive surgery [19]. Many studies have reported the reliability and reproducibility of CD measurements by Pentacam [20–22]. Lazaridis et al. [17] and Poyales et al. [11] applied this technique to explore CD changes after photorefractive keratectomy (PRK), laser-assisted in situ keratomileusis (LASIK), and SMILE in myopic eyes. Until now, changes in the CD profile after lenticule implantation and the effect of this change on efficacy of surgery are not well described.

We chose Pentacam to obtain and analyze CD changes in different corneal annular concentric zones and depths before and after SILI. The mean CD values of the total cornea were 16.60 ± 1.89 preoperatively, which is similar to the results of Lopes et al. [21]. When divided by depth, we found that the CD was highest at the anterior 120 μm layer compared to the central and posterior tissue layers before surgery, in accordance with the findings of Poyales et al. in myopic patients [11].

The results of our study showed that the densitometry of the total cornea significantly increased within the six months after SILI, especially in the anterior and mid-stromal layer. No change was found at the posterior layer of the cornea. These findings could be explained by the fact that, during the SILI procedures, the implanted lenticules were mainly located in the anterior 280 μm of the cornea (if SE = 10D), so that the CD of the posterior layer of the cornea was unaffected by the treatment. We hypothesize that the increase CD may be due to an increase of light backscattering between the cornea and lenticule interface as well as inflammatory responses after surgery. Previous studies have observed increases or decrease in corneal densitometry after corneal refractive surgery or cross-linking [8–11, 17, 22]. Different results may be attributed to different surgical procedures.

The characteristics of CD among different corneal annuli were also analyzed in the current study. The most pronounced change was found at the 0–2 mm annulus within three months postoperatively, while no difference was detected between the three annuli at six months after surgery. We speculate that the early postoperative corneal edema and inflammatory response may affect the central density of the corneal. As for the exact reason, further research is needed.

We additionally studied the changes of CD over time after surgery. The results showed that CD values did not increase progressively or regress to baseline, which suggests that corneal transparency is stable after SILI for hyperopia.

Previous studies have not demonstrated a correlation between increased corneal densitometry and visual acuity [9, 22, 23]. When we evaluated the possible relationship between the increase in CD and preoperative clinical parameters, no significant correlations were detected. Thus, we speculate that the increase in CD may be independent of the visual outcomes after SILI. Pradhan et al. found that the epithelium showed decreased thickness after SILI [5]. Similarly, our patients’ epithelial thickness show a downward trend over the postoperative period. However, the epithelial remodeling did not affect postoperative changes in CD.

There are several limitations of this study, namely, the lack of follow-up longer than 1 year, a relatively small sample size and the retrospective design. We will continue to enroll more patients and a longer follow-up period in the future to verify these findings.

In conclusion, this study reported CD changes of the cornea after the allogeneic corneal small-incision intrastromal lenticule implantation for the treatment of hyperopia. Our study found that SILI results in an increase in CD, particularly in the anterior and mid-stromal layer within the surgical zone of the cornea. However, the changes in CD did not correlate with the postoperative visual outcomes.

## Data Availability

The datasets used and/or analysed during the current study available from the corresponding author on reasonable request.
